# Genomic Regions Associated with Root Traits under Drought Stress in Tropical Maize (*Zea mays* L.)

**DOI:** 10.1371/journal.pone.0164340

**Published:** 2016-10-21

**Authors:** P. H. Zaidi, K. Seetharam, Girish Krishna, L. Krishnamurthy, S. Gajanan, Raman Babu, M. Zerka, M. T. Vinayan, B. S. Vivek

**Affiliations:** 1 International Maize and Wheat Improvement Centre (CIMMYT), Asia regional office, Hyderabad, India; 2 International Crops research institute for semi-arid tropics (ICRISAT), Hyderabad, India; Aberystwyth University, UNITED KINGDOM

## Abstract

An association mapping panel, named as CIMMYT Asia association mapping (CAAM) panel, involving 396 diverse tropical maize lines were phenotyped for various structural and functional traits of roots under drought and well-watered conditions. The experiment was conducted during *Kharif* (summer-rainy) season of 2012 and 2013 in root phenotyping facility at CIMMYT-Hyderabad, India. The CAAM panel was genotyped to generate 955, 690 SNPs through GBS v2.7 using Illumina Hi-seq 2000/2500 at Institute for Genomic Diversity, Cornell University, Ithaca, NY, USA. GWAS analysis was carried out using 331,390 SNPs filtered from the entire set of SNPs revealed a total of 50 and 67 SNPs significantly associated for root functional (transpiration efficiency, flowering period water use) and structural traits (rooting depth, root dry weight, root length, root volume, root surface area and root length density), respectively. In addition to this, 37 SNPs were identified for grain yield and shoot biomass under well-watered and drought stress. Though many SNPs were found to have significant association with the traits under study, SNPs that were common for more than one trait were discussed in detail. A total 18 SNPs were found to have common association with more than one trait, out of which 12 SNPs were found within or near the various gene functional regions. In this study we attempted to identify the trait specific maize lines based on the presence of favorable alleles for the SNPs associated with multiple traits. Two SNPs S3_128533512 and S7_151238865 were associated with transpiration efficiency, shoot biomass and grain yield under well-watered condition. Based on favorable allele for these SNPs seven inbred lines were identified. Similarly, four lines were identified for transpiration efficiency and shoot biomass under drought stress based on the presence of favorable allele for the common SNPs S1_211520521, S2_20017716, S3_57210184 and S7_130878458 and three lines were identified for flowering period water-use, transpiration efficiency, root dry weight and root volume based on the presence of favorable allele for the common SNPs S3_162065732 and S3_225760139.

## Introduction

In Asian tropics maize is largely (about 80%) grown as rain-fed crop, which is prone to face vagaries of monsoon rains associated with an array of abiotic and biotic constraints. Erratic/un-even distribution patterns of monsoon rain occasionally causes drought at different crop growth stage(s), which is identified as a factor responsible for year-to-year fluctuation in production of rainfed maize in Asian tropics[1]. Drought has been identified as the most important abiotic stress for the Asian region and addressing the problem of drought has been estimated to provide the highest technical returns to rainfed maize research and development investments in Asia [2]. While drought negatively affects all stages of maize growth and production, the reproductive stage particularly between tassel emergence and early grain filling, is the most sensitive to drought stress [3].

Past efforts in improving grain yield under water-limited conditions were accomplished by selecting for combination of traits such as increasing shoot biomass, shifting ratio between harvested grain vs shoot biomass, leaf surface area, number of tillers, earlier flowering etc. [4]. However, the real driving force of these above ground traits is root system and their functional properties that set the limits on shoot functions [5,6]. One of the strategies for improving yields in drought-prone rainfed system, when water availability in the root zone constrains crop growth, is to develop deeper and more profuse rooting system to access water from soil profile [7,8] and use the available water more efficiently (increasing water productivity) by increasing the water use efficiency [9,10]. Being first plant part that is exposed to various soil related stresses, including drought, roots play a pivotal role in adaptation to stress conditions and govern the overall performance of plants. Root characteristics are often assessed on the basis of surrogates (for example—leaf rolling), which may not accurately explain the stress-responsive (or adaptive) structural and functional changes in roots under stress conditions. Understanding how roots respond (or adjust) to stress conditions, and support adaptation to the stress is crucial for developing stress-resilient genotypes and there are several studies reported significant association between root traits and crop performance [11,12]. However, in spite of well-known role, in general and under drought stress conditions in particular, most often this important hidden-half is “knowingly” ignored due to complexity involved in studying root traits [1,13]. In recent years improvements of root traits to increase the efficiency of foraging the soil water and maintenance of productivity under drought and other abiotic stress is gaining momentum [5,14–16]. Keeping in view complexity in direct studies on root traits [17], the alternative for studying the available genotypic variability of such complex traits is to identify superior alleles through genome-wide association studies (GWAS), and use in forward breeding through marker-assisted introgression of desirable genomic regions in elite genetic background.

Association mapping differs from traditional linkage mapping that uses the ancestral recombination in natural populations to identify the marker-trait association based on linkage disequilibrium [18,19]. Effective association analysis to understand the inheritance of targeted traits can be performed with availability of abundant phenotypic variation and high density of polymorphism at the DNA sequence level. Maize, known for its abundant genetic divergence in nearly every trait of economic and agronomic importance and with the help of high density genotyping platforms like, Illimina ifinium and genotyping-by-sequencing (GBS) it is possible to develop millions of marker data points distributed throughout the genome for conducting an effective GWAS [20,21]. GWAS reports in maize are available for flowering time [22], kernel shape, 100 seed weight [23], kernel quality [24], functional mechanisms related to drought [25] and several other target genes for crop improvement [26,27]. Ample number of QTL mapping studies are available for several maize root traits [28–33], few GWAS studies were available for maize root traits in seedling stage [8] but to our knowledge no GWAS study is done for the root functional and structural traits at later growth stage. In this study, CIMMYT Asia association mapping (CAAM) panel was assembled using elite tropical and subtropical maize genotypes and phenotyped for root functional and structural traits under well-watered and drought stress conditions at reproductive stage to assess the available genotypic variability and to identify the significant genomic regions associated with root traits through GWAS studies, and their use in forward breeding through marker-assisted introgression of desirable genomic regions in elite genetic background.

## Results

### Phenotypic variation among root traits

Substantial variation (P<0.01) was observed among the CAAM panel lines for root functional traits under drought stress (DS) and well-watered condition (WW) and structural traits under drought stress. The variation was also significant among agronomic traits under the two water regimes (Table A in S1 File, Figure A and B in S2 File.). Broad-sense heritability for all the traits was high across moisture regimes (*h* >0.7), except anthesis silking interval (ASI) under well-watered condition (*h* = 0.41). In general, days to 50% male flowering (AD) was consistent across drought and well-watered conditions with a narrow variation of about one day. However, considerable variation was observed in days to 50% silking (SD) that delayed for >5 days or even no silk emergence in some lines under drought stress. Average grain yield per plant (GY) was higher (76.98 g/plant) under well-watered conditions, while the range was narrow under drought stress (0.02 to 108.10 g/plant) compared to well-watered condition (12.50 to 204.57 g/plant). Similar trend was also observed for shoot biomass (SB) with low values under drought stress (166.49 g/plant) as compared to well-watered condition (191.10 g/plant).

Among root functional traits, variation for both flowering period water use (WU) and transpiration efficiency (TE) was more wider under drought stress. WU was less (77.1%) under drought as compared to well-watered conditions. However, transpiration efficiency (TE) was higher (114.3%) under drought stress in comparison to well-watered conditions. Root structural traits under drought stress also revealed highly significant variation among the lines for all the traits (Table B in S1 File, Figure C in S2 File). Notably, the rooting depth (RD) ranged from 30.52 cm to 244.0 cm with the mean of 134.54 cm and the root dry weight (RDW) ranged between 0.84 g and 33.82 g with a mean of 10.63g.

A positive and significant relationship (p>0.001) was observed among structural and functional traits under drought stress condition, with an exception of root-length density (RLD), which seemed fairly independent of most of the functional root traits. Some of the noteworthy associations observed from the drought dataset were, i) between functional traits (WU and TE) and structural traits: root dry weight (RDW) [0.42 and 0.30], root volume (RV) [0.50 and 0.27] followed by rooting depth (RD) [0.27 and 0.27] and ii) between functional traits (WU and TE) and agronomic traits: grain yield (GY) [0.30 and 0.70], shoot biomass (SB) [0.60 and 0.99] and days to 50% male flowering (AD) [0.36 and 0.17] (Table C in S1 File). These strong positive associations observed under drought suggest that these traits, at least partly, might share common genomic regions. Under well-watered condition the root functional traits had significant positive correlation with grain yield and total plant biomass (Table C in S1 File).

### Molecular diversity and linkage disequilibrium

**Fig 1:**
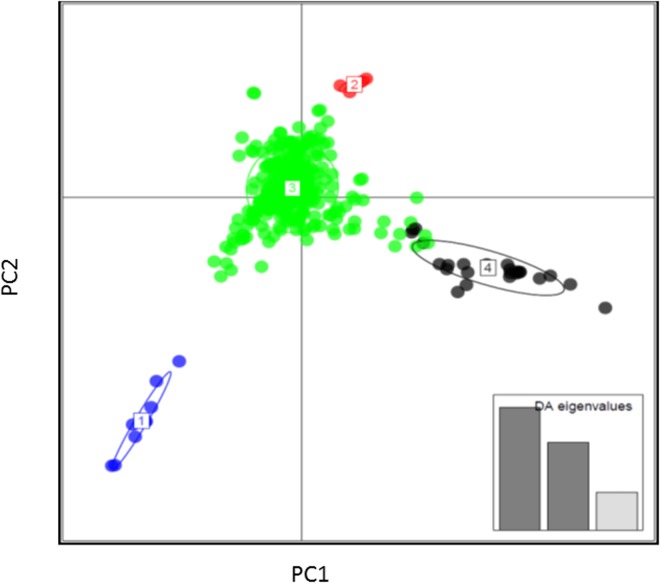
Clustering of CAAM panel based on discriminant analysis of principal component using the genetic data.

**Fig 2:**
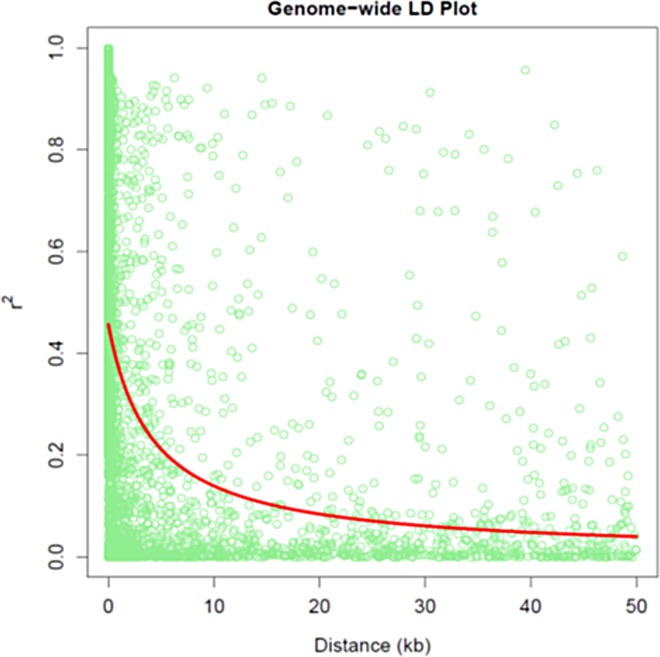
Linkage disequilibrium (LD) decay in the CAAM panel based on the SNPs

DAPC (Discriminant Analysis of Principal Components) analysis helped in establishing the population structure of the CAAM panel (Fig 1). DAPC was applied on the genetic data of the CAAM panel, which clustered these lines into four groups based on the first three PCs (Fig 1 Inset). The largest among the four group was group 3 with 345 lines predominantly with lines from lowland (64% of group and 91% of total panel) followed by lines from sub-tropical (23% of group and 89% of total panel). The groups 1, 2 and 4 were with less number of lines with mix of all adoption patterns. The whole genome linkage disequilibrium decay (LD) estimated from a random set of markers spanning across genome was observed at 5.6 kb (at r^2^ = 0.2) and 16 kb (at r^2^ = 0.1), with a general pattern of LD decay observed in most of the tropical maize germplasm (Fig 2). To improve the prediction accuracy and to eliminate the false positive associations, both PCA and the kinship matrix was used in the model.

### Mapping for functional root traits under well-watered condition

A total of 24 highly significant marker-trait associations (P = ≤10^−5^) were observed for the root functional traits [13 for WU and 11 for TE] under well-watered condition (Table D in S1 File). The phenotypic variation accounted for these traits by the SNPs ranged from 4 to 6% (Table 1). Interestingly several of these associations mapped to gene models that have been reported to have biological functions in response to stress or are components of photophosphorylation (Table D in S1 File). Among these associations, the following SNPs S3_210065070, S5_174937829, S5_206628645 and S7_141513891 are of particular importance for WU and SNPs S3_176556741, S3_225760139, S5_163230017, S6_158259266, S8_10392703, S9_113423602, S10_134418000 for TE, in lieu of their i) direct association with the trait of interest and ii) close association with gene models associated with stress response or water use traits

**Table 1:** Summary of Marker trait association (MTAs) under well-watered (WW) and drought stress condition (DS). **W**- Well watered condition, **DS**- Drought stress condition, **GY**- Grain yield, **SB**- Shoot biomass, **WU**- Flowering period water use, **TE**- Transpiration efficiency, **RD**-Rooting depth, **RDW**-Root dry weight, **RL**-Root length, **RSA**-Root surface area, **RV**- Root volume, **RLD**- Root length density, **PV**- phenotypic variation.

Traits	No. of MTAs under WW	No. of MTAs under DS	Total No. of MTAs	P-Value range	Range of PV explained
GY	15	13	28	10^−6^ to 10^−05^	0.04 to 0.06
SB	10	12	22	10^−6^ to 10^−05^	0.04 to 0.06
WU	13	13	26	10^−6^ to 10^−05^	0.04 to 0.06
TE	11	13	24	10^−6^ to 10^−05^	0.04 to 0.06
RD		13	13	10^−8^ to 10^−05^	0.04 to 0.05
RDW		11	11	10^−7^ to 10^−05^	0.05 to 0.07
RL		10	10	10^−7^ to 10^−06^	0.05 to 0.07
RSA		11	11	10^−7^ to 10^−06^	0.05 to 0.07
RV		11	11	10^−08^ to 10^−06^	0.05 to 0.07
RLD		11	11	10^−6^ to 10^−05^	0.04 to 0.06
**Total**	**49**	**118**	**167**		

### Mapping for functional and structural traits under drought stress condition

Under drought stress a total of 26 significantly associated SNPs were identified for WU (13 SNPs) and TE (13 SNPs) (Table 1). The phenotypic variation explained by these SNPs ranged between 4 and 6%. The SNPs, S1_256693112, S2_150015274, S3_140832594, S3_162065732, S4_177880350, S8_1677226454, S8_2079947, S9_117308991 associated with WU were found in different gene models GRMZM2G100448, GRMZM2G31470, GRMZM2G089638, GRMZM2G180406, GRMZM2G300624, GRMZM2G350020, GRMZM2G417125 and GRMZM2G117956 and the SNPs, S1_211520521 (GRMZM2G131205), S1_295003474 (GRMZM2G138382), S5_187657126 (GRMZM2G120922), S7_137170071 (GRMZM2G429118) and S7_17332809 (GRMZM2G349655) associated with TE were found in different gene model had the biological functions related to various drought tolerant mechanisms like osmotic adjustment, signaling pathways etc. (Table D in S1 File).

For root structural traits a total of 67 significantly associated (P = ≤10^−5^) SNPs were identified:13 SNPs for rooting depth (RD), 11 SNPs for root dry weight (RDW), root surface area (RSA), root volume (RV) and root-length density (RLD) and 10 SNPs for root length (RL). These SNPs accounted for the variation of 4 to 8% for these structural traits. In specific SNPs, S1_285063931, S1_119655560, S3_219690579, S5_190243480, S10_25666760 associated with RD; SNPs S9_131567840 and S9_151573444 associated with RL, and one SNP S5_206615952 associated with RSA; S2_43559512, S2_198898603, S3_162065732 associated with RDW were found within the different gene models with various biological functions like stress signaling, stress related protein and ion transport absorption and regulation of stress related transcription factors.

### Common association for agronomic and root traits

**Fig 3:**
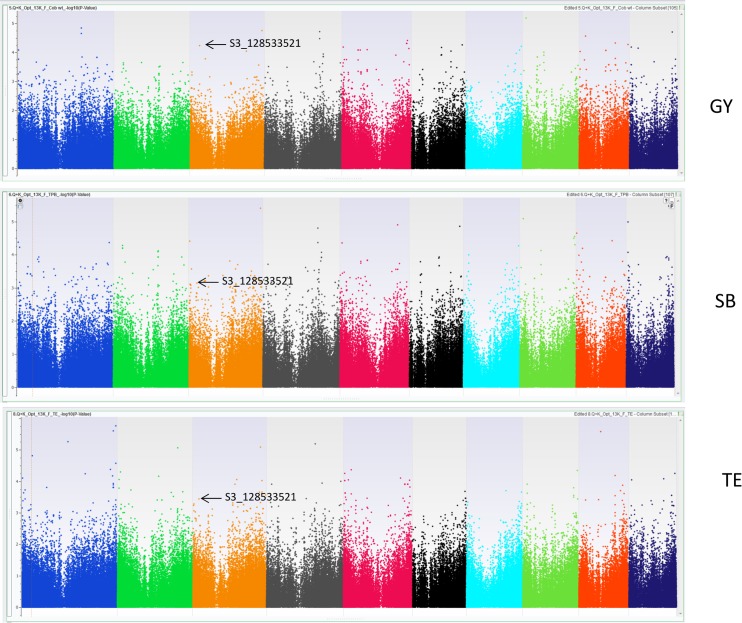
Genome wide association scans for markers- S3_128533521 and S7_151238865 associated with Grain yield (GY), Shoot biomass (SB) and Transpiration efficiency (TE) under well water condition.

Though several SNPs (167 SNPs) showed significant association, SNPs that were common for more than one trait gains more important from breeding point of view. In present study three SNPs (S3_12853321, S3_176556741 and S7_151238865), each commonly associated with grain yield (GY), shoot biomass (SB) and TE (Fig 3) and four SNPs (S2_140892540, S3_225760139, S5_59423673 and S8_10392703) each were found to be associated with SB and TE under well-watered conditions (Table 2). Under drought stress 9 SNPs each were common between TE and SB. Out of these 9 SNPs, 8 were found within different gene model (Table 2 and Table D in S1 File).

**Table 2:** List of Significant SNPs associated with more than one functional and structural trait under drought stress (DS) and well-watered condition (WW). **WW**- Well watered condition, **DS**- Drought stress condition, **GY-** Grain yield, **SB**- Shoot biomass, **WU**- Flowering period water use, **TE**- Transpiration efficiency, **RDW**-Root dry weight, **RV**- Root volume, **PVE**- phenotypic variation, **Chr**- Chromosome Number, **MAF**-Minor allele frequency

S.No	Trait name	Condition	SNP	Chr	Position (Mb)	Favorable allele	MAF	MLM P-Value	PVE	Gene Name
1	GY + SB	WW	S2_215946315	2	215.95	T	0.14	1.2x10^-05^	0.05	
2	GY + SB + TE	WW	S3_128533521	3	128.53	A	0.05	6.5x10^-06^	0.05	
3	GY + SB + TE	WW	S3_176556741	3	176.56	T	0.12	2.7x10^-05^	0.05	GRMZM2G426108
4	GY + SB + TE	WW	S7_151238865	7	151.24	G	0.38	3.2x10^-05^	0.05	
5	SB + TE	WW	S2_140892540	2	140.89	T	0.04	1.5x10-^05^	0.05	
6	SB + TE	WW	S5_59423673	5	59.42	A	0.15	2.2x10^-06^	0.06	
7	SB + TE	WW	S8_10392703	8	10.39	G	0.07	3.4x10^-06^	0.06	GRMZM2G374085
8	SB + TE	DS	S1_21984561	1	21.98	C	0.24	3.6 x 10^−05^	0.05	GRMZM2G388915
9	SB + TE	DS	S1_211520521	1	211.52	G	0.18	3.5 x 10^−06^	0.06	GRMZM2G131205
10	SB + TE	DS	S2_20017716	2	20.02	T	0.16	8.2x10^-05^	0.05	
11	SB + TE	DS	S3_57210184	3	57.21	T	0.07	2.9x10^-06^	0.06	GRMZM2G331811
12	SB + TE	DS	S5_18624158	5	18.62	T	0.06	4.9x10^-05^	0.04	GRMZM2G363437
13	SB + TE	DS	S5_187657126	5	187.66	A	0.11	4.5x10^-05^	0.04	GRMZM2G120922
14	SB + TE	DS	S7_130878458	7	130.88	T	0.38	5.4x10^-06^	0.05	AC198894.4_FGT002
15	SB + TE	DS	S7_137170071	7	137.17	C	0.03	9.6x10^-06^	0.05	GRMZM2G429118
16	SB + TE	DS	S7_155619721	7	155.62	C	0.04	2.3x10^-05^	0.05	GRMZM2G158009
17	WU + RDW	DS	S3_162065732	3	162.07	G	0.17	3.2x10^-05^	0.05	GRMZM2G180406
18	SB + TE + RV	DS	S3_225760139	3	225.76	G	0.19	1.9x10^-06^	0.06	GRMZM2G369956

**Fig 4:**
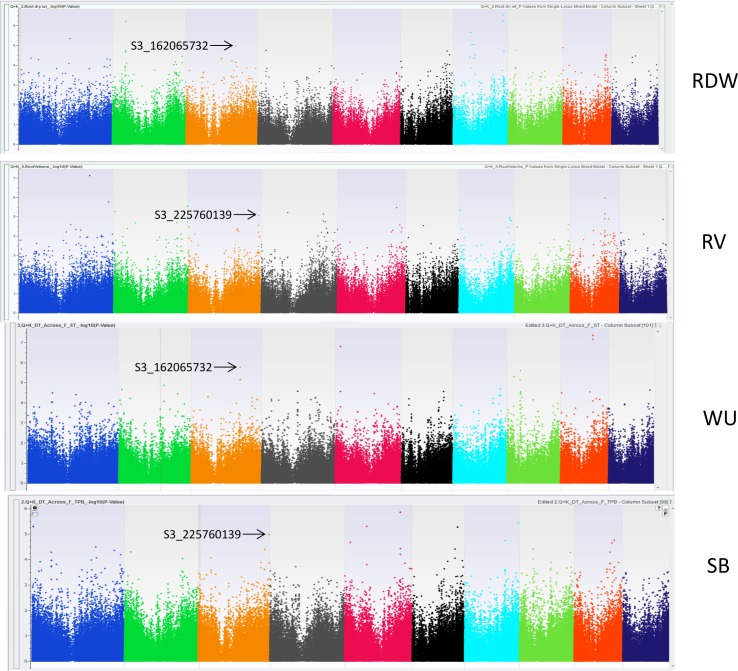
Genome wide association scans for markers- S3_162065732 associated with root dry weight (RDW) and flowering period water use (WU) and S3_225760139 associated with root volume (RV) and Shoot biomass (SB) under drought stress condition.

Among the SNPs associated with root structural traits two SNPs each located on chromosome 3 (S3_162065732 and S3_225760139) were observed common between WU and RDW and between SB, TE and RV, respectively (Fig 4). Among these associations for functional and structural traits, favorable associations have been largely observed in chromosome 3 suggesting the presence of either a common gene or gene clusters responsible for drought stress tolerance in maize.

## Discussion

Breeding for drought tolerance is one of the priority traits for most of the maize breeding programs in tropics. In general, drought tolerance depends on root structural traits for extracting the water and nutrient from soil [34,35] and root functional traits for effective utilization of available water [36]. The success of breeding for drought tolerance is limited with conventional breeding alone, especially in the present condition of rapid and catastrophic changes in weather patterns [20]. Integration of advances in genomics tools and resources with conventional breeding might improve the ability to identify the desirable unique or rare alleles from the germplasm collection [37]. In the present study, GWAS was carried on CAAM panel, which was genotyped using GBS platform and phenotyped for root structural and functional traits under drought and well-watered condition. Exploiting this variability and identifying significant marker-trait associations based on GWAS might support breeding for drought tolerant maize through marker-assisted introgression of key genomic regions related to promising root traits into elite germplasm.

Phenotypic variation for root functional traits was significantly high under both well-watered and drought stress conditions. The range of WU was wider, while it was narrow in case of TE under drought stress in comparison to well-watered condition. Under well-watered conditions the cylinders were maintained at optimal moisture (at field capacity), all the genotypes transpire at similar rate without significant variation. However, under drought stress, the rate of transpiration and biomass production varied significantly depending upon drought tolerance among genotypes and water-use pattern. The range various traits indicated that there is substantial variation exists among maize germplasm for these traits. High broad-sense heritability (*h*>0.5) for both functional and structural traits indicated that these traits are highly repeatable. Similar heritability values for roots traits under controlled and field conditions at various growth stages were also reported in previous studies [8,38,39]. High heritability for these highly dynamic traits might be due to the fact that the experiment was conducted in semi-controlled condition and also because of high accuracy of the root image analysis software. Relationship between root functional and structural traits with the grain yield suggested that these traits play significant role in overall plant performance of genotypes, including final grain yields under drought stress.

In general, larger LD block and slower rate of LD decay results in low mapping resolution. In diverse maize germplasm the LD decay occurs rapidly within few kilo-base pairs due to high rate of recombination [40–42]. In our study we obtained LD decay of 5.6 kb (r^2^ = 0.2) in CAAM panel using GBS data on sub-tropical and tropical lines. Several studies using GBS for large number of tropical and temperate maize germplasm indicated that higher mapping resolution can be obtained from tropical germplasm [21] because LD decay is faster in tropical germplasm than in temperate germplasm [43,44].

In present study, a total of 50 SNPs for root functional traits, 67 SNPs for root structural traits and 28 SNPs were identified for grain yield and shoot biomass under well-watered and drought stress condition. Though several individual SNPs are associated with various root functional and structural traits, some of the SNPs were within various gene models, which had biological functions related to drought tolerance mechanism. Genes found to have significant associations with target traits could be re-sequenced in a diverse panel of germplasm to identify causal mutations and the most favorable alleles for trait improvement and to develop simple PCR-based markers for MAS [45,46].

The SNPs S1_252377015 associated with grain yield under well-watered, S3_162065732 associated with WU and RDW under drought stress and S5_174937829 associated with WU under well-watered, were mapped with in the bHLH loci viz., bHL43, bHLH 152 and bHLH 32, respectively. Many cellular processes and responses that are important for plant to tolerate various abiotic stresses were controlled by this bHLH, which is a large family of conserved transcription factors [47]. This has also been known to interact with two other drought-tolerant genes, *bZIP* (regulates auxin transport) [48] and *MYB* light signaling transductions, including photosynthesis in *Arabidopsis thaliana* [49]. The SNP S9_117308991 associated with WU under drought stress was mapped at 0.102kb away from the gene model GRMZM2G117956, which has the biological function of proline catabolic process. Proline, a “compatible solute” acts as energy sources during stress conditions and plays a diverse role in stabilizing protein, membranes, subcellular structures and protecting cellular functions by scavenging reactive oxygen species [50]. Accumulation of proline under stress condition helps in changing the carbon flux through oxidative pentose phosphate pathway (OPPP) that leads to synthesis of nucleotides and in turn accelerates cell division upon relieve of stress [51].

The SNPs S3_140832594, S4_25790364, S5_190243480 associated with WU, RDW and RD under drought stress was co-localized with gene models associated with G-protein coupled receptor signaling pathway. The static nature of plants evolved them to have an efficient system to respond to the environment fluctuation at membrane level through signal generation and transduction. Among those the G-protein coupled signal transduction plays a major role [52]. G-proteins are involved in ABA-induced stomatal movements by controlling inward and outward-rectifying potassium current or an anion channel [53], which in turn determines the stomatal aperture size by maintaining the guard cell turgor pressure. These individual SNPs which were associated with various root functional and structural traits found within loci or close to loci associated with various drought tolerance and avoidance mechanisms could be developed as gene based markers. Gene-based markers are more accurate than linked markers for the prediction of phenotype, since the marker–trait association will not be lost during segregation in the course of recurrent breeding selection cycles [42].

Maximum number of SNPs were found on the chromosome 5 (9 SNPs), followed by chromosome 3 and 7 (8 SNPs each) for the root functional traits and for structural traits maximum number of SNPs were found on Chromosome 1 and 9 (9 SNPs each) followed by chromosome 2 and 7 (8 SNPs each) under well-watered and drought stress condition. On chromosome 7 at 123.61 to 132.68 Mb and on chromosome 3 at 169.75 to 178.23Mb were reported to be the regions associated with drought tolerance or drought adaptiveness as they harbor 5 and 6 meta QTLs for grain yield and ASI, respectively under well-watered and drought stress condition [54]. In the present study on chromosome 7 and 3 the SNPs S7_130878458 at 130.88Mb and S7_13717001 at 137.17 Mb associated with WUE and SB under drought stress, S7_132080112 at 132.08Mb associated with RSA, S7_133377796 at 133.38Mb associated with RD, S3_176556741 associated with TE, GY and SB were found in those meta QTL regions. Similarly, the SNPs associated with root structural traits found on the chromosome 1 were also found in the meta QTL region [55]. Identification of these SNPs associated with root functional and structural traits within QTL regions related to grain yield under well-watered and drought stress conditions indicates the contribution of those root traits in grain yield under drought stress. This suggests that the SNPs identified in these regions could be used in drought tolerance breeding for screening and selecting drought tolerant trait donor lines with high yield.

Results from association analysis could be used to predict the best haplotypes across one or multiple genes for optimum expression of the target trait. Association analysis can help to determine which one is the best donor, something that linkage analysis cannot, as donors vary in their background effects in terms of the effects of alleles at other loci that directly or indirectly influence the target trait [19]. In this study we attempted to identify trait specific maize lines based on the presence of favorable alleles for the SNPs associated with multiple traits (Tables 2 and 3). Seven lines were identified for transpiration efficiency, shoot biomass and grain yield under well-watered conditions based on the favorable allele for two SNPs S3_128533512 and S7_151238865, four lines were identified for transpiration efficiency and shoot biomass under drought stress based on the presence of favorable allele for the common SNPs S1_211520521, S2_20017716, S3_57210184 and S7_130878458 and three lines were identified for flowering period water-use, transpiration efficiency, root dry weight and root volume based on the presence of favorable allele for the common SNPs S3_162065732 and S3_225760139. These selected lines and SNPs associated with multiple traits and various gene models could be used as trait donor lines for root function and structural traits after validating them in field conditions.

**Table 3:** Accessions selected possessing desirable allelic combination for multiple traits and there phenotypic expressions under DS and WW condition. **WW**- Well watered condition, **DS**- Drought stress condition, **GY**- Grain yield, **SB**- Shoot biomass, **TE**- Water use efficiency, WU-Flowering period water use **RDW**-Root dry weight, **RV**-Root volume.

S.No	Trait and selected accessions	Associated SNP	DS				WW			
	GY + SB + TE under WW		WU	TE	SB	GY	WU	TE	SB	GY
1	CL02450-B*5	S3_128533521,S7_151238865	13.3	3.6	200.6	61.2	17.4	4.2	318.0	125.5
2	(CLQ-RCYQ46 = (CML150xCL-03618)-B-17-2-2-BxCL-RCY017)-B-23-2-BB-2-B*5		13.8	3.3	176.0	78.1	16.8	3.3	242.2	116.6
3	CML317-2-BBB		14.0	3.2	170.8	75.1	18.1	3.1	227.5	112.0
4	90[SPMATC4/P500(SELY)]#-B-48-4-B*7		15.6	5.0	269.5	101.6	18.6	4.6	307.8	124.9
5	CML284-2-BBB		12.8	4.6	256.8	86.9	18.2	3.5	259.3	121.0
6	CML227-B*6		11.1	3.5	183.2	69.9	19.5	4.2	335.3	127.9
7	WLS-F191-2-1-1-B-1-B*4		13.5	3.4	178.2	71.1	15.8	4.7	327.5	118.0
	**SB + TE Under DS**									
8	[M37W/ZM607#bF37sr-2-3sr-6-2-X]-8-2-X-1-BBB-xP84c1F27-4-3-3-B-1-B]F29-1-2-2x[KILIMAST94A]-30/MSV-03-101-08-BB-1xP84c1F27-4-1-4-B-3-B]F2-1-2-1-1-1-BxCML486]-1-1-BB	S1_211520521,S2_20017716,S3_57210184,S7_130878458	14.7	5.5	284.9	97.8	20.1	4.6	310.3	77.3
9	CML452 = Ac8328BNC6-166-1-1-1-B*12		16.8	3.9	214.5	65.2	17.4	3.2	226.0	85.5
10	CML486 = P45c8-76-1-2-1-2-B*12		11.7	3.4	185.0	67.8	17.0	3.3	242.3	106.4
11	CA00102/CA00106-B-12-2-B*4		12.2	3.3	171.6	65.2	17.6	3.1	206.5	57.3
	**SB + TE under WW**									
12	CML181-B*5	S2_140892540,S5_59423673,S8_10392703	14.1	3.2	179.4	46.6	17.4	4.3	317.3	113.5
13	DTPYC9-F46-1-2-1-1-B*4		13.1	3.5	186.0	86.3	16.5	3.3	217.8	111.8
	**WU+ TE + RDW + RV Under DS**		WU	TE	RDW	RV				
14	CL-RCY023 = (CL-02439*CML286)-B-1-2-2-B*10	S3_162065732,S3_225760139	13.5	4.0	13.24	81.54	18.1	3.7	252.8	106.9
15	CML171-BBB-1-B*5		14.8	3.2	20.61	176.37	17.5	3.2	243.3	106.9
16	CLRCY030-B*5		11.2	3.2	15.41	123.60	16.1	3.7	295.6	161.0

## Conclusion

The SNPs identified for root traits, directly or indirectly related with drought tolerance mechanism, might help in selecting trait-specific donor lines or lines with favorable allele for multiple traits. These genes uncover physiological responses and molecular mechanisms related to drought tolerance. Genes governing several functional traits were identified, including stomatal closure, reduced water potential, root development, signaling pathways. In addition, breeding approach using this information upon validation will greatly help to increase our understanding of the genetic architecture of complex traits under drought stress condition. The major genomic regions with favorable alleles for key roots traits associated with drought tolerance can be introgressed into elite and locally adapted genetic background through step-wise marker-assisted validation-cum-introgression strategy.

## Material and Methods

### Germplasm

An association mapping panel, named as CIMMYT Asia association mapping (CAAM) panel, involving 396 diverse tropical maize lines (Table E in S1 File) were phenotyped for various structural and functional aspects of roots. The panel was constituted by involving selected advanced stage maize inbred lines derived from CIMMYT’s tropical and sub-tropical pools and populations from Latin America, Africa and Asian maize program. The 396 lines were selected out of over 1000 lines evaluated in Asian tropics for their general adaptation under optimal growing conditions. The lines with reasonably good adaptation in Asian tropics were selected for constituting the CAAM panel, avoiding sister lines or over-representation of lines derived from any specific pools or populations. Apart from lines from CIMMYT-Asia maize program, the panel included the lines derived from CIMMYT’s drought tolerant populations, including *Tuxpeno Sequia*-C6 (Tropical late white-dent), *La Posta Sequia*-C7 (Tropical late white-dent), DTPY-C9 (Tropical medium yellow-flint), Pool-26 Sequia (Tropical late yellow-flint), DTPW-C9 (Tropical medium white-flint), G18 *Sequia* C5 (Tropical early yellow-dent) and Pool16 BN *Sequia*-C5 (Tropical medium white-dent), which were systematically developed and improved for drought tolerance through full-sib or S1 recurrent selection scheme [56]. The panel was phenotyped for various root traits, including root functional traits such as–flowering period water use, transpiration efficiency under both drought and well-watered condition, and for root structural traits, including rooting depth, root dry weight, root length, root volume, root surface area and root length-density under drought stress. Phenotyping was done during 2012 and 2013 *Kharif* (summer-rainy) season. During 2012 *Kharif* the panel was phenotyped under drought stress for root functional traits with four replications and for root structural traits with two replications. During 2013 *Kharif* the panel was phenotyped for root functional traits under drought and well-watered conditions with four and two replications, respectively. In both the seasons the experiment was laid-out in RCBD.

### Root trait phenotyping

The experiments were conducted in root phenotyping facility at CIMMYT-Hyderabad, India (17.3850° N, 78.4867° E and 505 masl), which is based on the lysimetric system that provides opportunity to directly assess and quantify root traits and their dynamics under various growing conditions and allows high-precision phenotyping of various root traits. It facilitated the root study based on a real-time measurement of water uptake, water use and an assessment of variation in root structural traits under different growing condition in the rhizosphere. Plants were grown in mini-rhizotrons, which was made-up of PVC (Polyvinyl chloride) tube of 25 cm diameter and 150 cm length, filled with a mixture of Vertisol + Alfisol + sterilized farm yard manure in the ratio of 15:5:1 by volume. A PVC end plate was retained at 3.0 cm from the bottom with four screws in a way to retain the soil at the bottom but to freely allow water drainage. The cylinders had a very similar bulk density to field conditions, close to 1.27, and cylinders weighed a mean weight of 172 kg. The soil that was used to fill the cylinders was thoroughly incorporated with ammonium sulfate, urea, muriate of potash and zinc sulphate at the rate of 800, 174, 320 and 53 mg kg^-1^ soil. A top dressing of 3.0 g urea cylinder^-1^ was done at 30 and 50 days after sowing.

The PVC tubes were arranged in eight trenches (1.5 m deep, 2.0 m wide and 25 m long) in a way to match levels of cylinder and outside soil surfaces and separated from one another by a distance of approximately 20.0 cm. In this way maize plants were placed at a density of 6.25 plants m^-2^, matching with plant population close to a field planting density (row-to-row distance of 60.0 cm and plant-to-plant spacing of 20.0 cm). Border effects were managed by placing a row of potted plants on all sides of the trenches. Weighing of the cylinders was done by lifting the cylinders using the metal-collar fixed at the top of the cylinder, with a block-chained pulley. A S-type load cell (Mettler-Toledo, Geneva, Switzerland) was hooked between the collar of the cylinder and the pulley. A scale of 200 kg capacity allowed repeated measurements with accuracy of 20.0 g.

### Trial management and stress treatment

In each cylinder three seeds were sown and a measured amount of water (5.0 liters cylinder^-1^) was applied as 1^st^ irrigation for germination, and 10 days after sowing thinning was done to maintain one seedling per cylinder. Metrological parameters such as minimum, maximum temperature, evaporation and rainfall were recorded on daily basis from date of sowing until the crop harvest (Table F in S1 File). GDD (Growth degree days) was recorded starting from date of sowing until the GDD is reaching 550°C. Till that stage a measured amount of water was applied to maintain optimal moisture conditions in each cylinder. At 550°C GDD each cylinder was saturated with 25.0 liters of water, and there after irrigation was stopped in cylinders labeled for drought stress treatment, whereas application of measured amount of water was continued in cylinders labeled for well-watered treatment. In both the treatments soil surface of the cylinders was covered with polyethylene beads to prevent evaporative loss of moisture from the soil in cylinders. In this way it was ensured that water loss from the cylinder was largely through the transpiration process by the plants. Once the excess water flow stopped after full-saturation (about after 24hrs) each cylinder were weighed in both the treatment, which was noted as initial weight of the cylinder. In well-watered treatment the moisture was maintained by weighing the cylinders on regular intervals and the amount water lost from the initial weighing was compensated by adding the loss of water through transpiration process. However, in drought treatment no irrigation was applied until two weeks after anthesis. This was followed on individual genotype basis in drought stress treatment, i.e. resuming irrigation two weeks after completion of male flowering. Before resuming irrigation in drought treatment a final weight of the cylinder was recorded. Simultaneously, final cylinder weight for respective genotype was also recorded under well-watered conditions as well. The difference in initial weight and final weight were used in calculating the amount of water used during flowering period under drought stress or well-watered conditions.

### Phenotyping root traits

Total amount of water used was accounted until the physiological maturity and the plant biomass was also recorded by harvesting the whole plants (excluding roots). The root functional traits were calculated as follows:

Floweringperiodwateruse(WUL)=Intialweightofthecylinder−finalweightofthecylinder

$$Transpiration\;efficiency\;(TE\;gL^{-1})=\frac{Shoot\;biomass\;(g)}{Amount\;of\;water\;transpired\;(L)}$$

For measuring the root structural traits, cylinders were shifted to root washing area and adhering soil around the root was carefully removed by passing a fine-jet of water through the cylinder. After removing the soil, the intact root was taken out of cylinder and washed once again with water to remove small solid clades adhered to the roots. Rooting depth (RD) was measured as the length of root from stem collar to tip of the root. Roots were scanned and digitalized as images for measuring the root volume (RV), root surface area (RSA) and root length (RL) and root length density (RLD) using Shimadzu scanner and analyzing with Winrhizo software (Winrhizo, Regent Ltd, Canada). Roots were then dried in hot-air oven at 70°C for three days and root dry weight was recorded. In addition to root functional and structural traits the plant traits such as—anthesis date, silking date, anthesis-silking interval (ASI) and grain yield were also recorded in all the experiments.

### Genotype data

A total of 955, 690 SNPs were generated through GBS v2.7 using Illumina Hi-seq 2000/2500 at Institute for Genomic Diversity, Cornell University, Ithaca, NY, USA. The physical coordinates of GBS SNPs was derived from AGPv2. The GBS service provider (Institute for Genomic Diversity, Cornell University, Ithaca, NY, USA), imputed the missing data points by performing a partial imputation based on an algorithm that searched for the closest neighbor in small SNP windows across the entire maize database (~22,000 Zea samples) allowing for 5% mismatch. The criteria for filtering SNPs for GWAS, PCA and LD analysis were done based on Suwarno *etal*., 2015 [57] with slight modifications. Based on criteria of call rate (CR) >0.7 and with minor allele frequency (MAF) > 0.03, we obtained 331, 390 SNPs from the total SNPs for association analysis. The marker density for the present association mapping panel was 1 SNP per 6.209Kb with minimum gap of 1bp and maximum of 1763.22kb. From this 331,390 SNPs a subset of 71, 595 high quality SNPs, were filtered by increasing the filtering stringency to CR>0.95 and MAF>0.1, which were used to derive PCA and Kinship matrices.

### Statistical analysis of phenotype data and association analysis

Analysis of variance of root functional and structural traits along with other agronomical traits was done separately for each year. A combined analysis over the years was done for root functional traits under drought stress condition. Mean values of functional (well water and drought) and structural traits (drought) was used to estimate the pearson correlation coefficient and descriptive statistics using Genstat 14^th^ edition [58]. The mean values were used for GWAS analysis using SNP and Variation Suits v8.**x** (Golden Helix, Inc., Bozeman, MT, www.goldenhelix.com)[59].

The population structure of the panel was evaluated by performing discriminant analysis of principal component (DAPC) using the ‘adgenet’ library in R software. The extent of genome-wide linkage disequilibrium was estimated based on adjacent pairwise r^2^ values and the physical distance among the SNPs using ‘nlin’ function in R. To perform mixed linear model (MLM) based association analysis, Principal component analysis and kinship matrix analysis were carried out using SVS. The models used in the association study were based on visual observation of the quantile-quantile (Q-Q) plots (Figure D in S2 File.), which was the plots of observed–log10 P values versus expected–log10 P values under the null hypothesis that there is no association between marker and the phenotype[21]. To identify the significant GWAS signals multiple criteria filtering options was used. Top 10 to 15 SNPs were selected based on smallest p value (<10^−4^), phenotypic average for homozygous minor allele genotype (DD) (greater or smaller than average based on trait) and presence of rare allele in more than 11 genotypes (3% of population).

## Supporting Information

S1 File Table A in S1 File. Descriptive statistics for root functional traits and agronomical traits under well-watered (WW) and drought stress (DS) condition. Table B in S1 File. Descriptive statistics for root structural traits under drought stress condition. Table C in S1 File. Correlation coefficient for the agronomical, root functional (drougt stress and well-watered condition) and structural (Drought stress) traits of CAAM panel. Table D in S1 File. Marker trait assoication for functional and structural traits under well-watered (WW) and Drought stress condition (DS). Table E in S1 File. Pedigree details of CIMMYT Asia Association Mapping (CAAM) panel. Table F in S1 File. Meteorological information for year 2012 and 2013 during the experiment period.
(XLSX)

S2 File Figure A in S2 File. Frequency distribution of grain yield, total plant biomass, transpiration efficiency and flowering period water use under well water condition. Figure B in S2 File. Frequency distribution of grain yield, total plant biomass, transpiration efficiency and flowering period water use under drought stress condition. Figure C in S2 File. Frequency distribution of root structural traits under drought stress condition. Figure D in S2 File. Q-Q plots of various model for root functional traits under well-watered and drought stress condition and for root structural traits under drought stress condition.
(XLSX)
